# A variant in the MICA gene is associated with liver fibrosis progression in chronic hepatitis C through TGF-β1 dependent mechanisms

**DOI:** 10.1038/s41598-018-35736-2

**Published:** 2019-02-05

**Authors:** Rasha El Sharkawy, Ali Bayoumi, Mayada Metwally, Alessandra Mangia, Thomas Berg, Manuel Romero-Gomez, Maria Lorena Abate, William L. Irving, David Sheridan, Gregory J. Dore, Ulrich Spengler, Pietro Lampertico, Elisabetta Bugianesi, Martin Weltman, Lindsay Mollison, Wendy Cheng, Stephen Riordan, Rosanna Santoro, Rocío Gallego-Durán, Janett Fischer, Jacob Nattermann, Roberta D’Ambrosio, Duncan McLeod, Elizabeth Powell, Olivier latchoumanin, Khaled Thabet, Mustafa A. M. Najim, Mark W. Douglas, Christopher Liddle, Liang Qiao, Jacob George, Mohammed Eslam, International Liver Disease Genetics Consortium (ILDGC), Rose White, Angela Rojas, Margaret Bassendine, Chiara Rosso, Lavinia Mezzabotta, Reynold Leung, Barbara Malik, Gail Matthews, Jason Grebely, Vincenzo Fragomeli, Julie R. Jonsson

**Affiliations:** 1 Storr Liver Centre, The Westmead Institute for Medical Research and Westmead Hospital, University of Sydney, and Westmead Hospital NSW, Sydney, Australia; 2 Division of Hepatology, Ospedale Casa Sollievo della Sofferenza, IRCCS, San Giovanni Rotondo, Italy; 3 Section of Hepatology, Clinic for Gastroenterology and Rheumatology, University Clinic Leipzig, Leipzig, Germany; 4 Unit for The Clinical Management of Digestive Diseases and CIBERehd, Hospital Universitario Virgen del Rocío, University of Seville, Sevilla, Spain; 5 Division of Gastroenterology and Hepatology, Department of Medical Science, University of Turin, Turin, Italy; 6 NIHR Biomedical Research Unit in Gastroenterology and the Liver, University of Nottingham, Nottingham, United Kingdom; 7 Institute of Translational and Stratified Medicine, Plymouth University, Plymouth, United Kingdom; 8 Kirby Institute, The University of New South Wales, Sydney, NSW Australia; 9 Department of Internal Medicine I, University of Bonn, Bonn, Germany; 10 Università degli Studi di Milano Centro A.M. e A. Migliavacca, First Division of Gastroenterology, Fondazione IRCCS Ca’ Granda Ospedale Maggiore Policlinico, Department of Pathophysiology and Transplantation Milan Italy, Milan, Italy; 11 Department of Gastroenterology and Hepatology, Nepean Hospital, Sydney, NSW Australia; 12 Department of Gastroenterology and Hepatology, Fremantle Hospital, Fremantle, WA Australia; 13 Department of Gastroenterology & Hepatology, Royal Perth Hospital, Wellington, WA Australia; 14 Gastrointestinal and Liver Unit, Prince of Wales Hospital and University of New South Wales, Sydney, NSW Australia; 15 Department of Anatomical Pathology, Institute of Clinical Pathology and Medical Research (ICPMR), Westmead Hospital, Sydney, Australia; 16 The University of Queensland, School of Medicine, Princess Alexandra Hospital, Woolloongabba, QLD Australia; 17 Biochemistry Department, Faculty of Pharmacy, Minia University, Minia, Egypt; 18 Department of Medical Laboratories Technology, Faculty of Applied Medical Sciences, Taibah University, Medina, Saudi Arabia; 19 Centre for Infectious Diseases and Microbiology, Marie Bashir Institute for Infectious Diseases and Biosecurity, University of Sydney at Westmead Hospital, Westmead, NSW Australia; 20 Institute of Translational Medicine, Newcastle University, Tyne, UK

## Abstract

Hepatocarcinogenesis is tightly linked to liver fibrosis. Recently, two GWAS variants, *MICA* rs2596542 and *DEPDC5* rs1012068 were identified as being associated with the development of HCV-induced hepatocellular carcinoma (HCC) in Japanese patients. The role of these variants on hepatic inflammation and fibrosis that are closely associated with HCC development is not known, nor are the biological mechanisms underlying their impact on the liver. Here, we demonstrate in 1689 patients with chronic hepatitis C (CHC) (1,501 with CHC and 188 with HCV-related HCC), that the *MICA* (T) allele, despite not being associated with HCC susceptibility, is associated with increased fibrosis stage (OR: 1.47, 95% CI: 1.05–2.06, p = 0.02) and fibrosis progression rate (hazards ratio: 1.41, 95% CI: 1.04–1.90, p = 0.02). The *DEPDC5* variant was not associated with any of these phenotypes. MICA expression was down-regulated in advanced fibrosis stages. Further, (T) allele carriage was associated with lower MICA expression in liver and serum. Transforming growth factor-β1 (TGF-β1) expression suppresses MICA expression in hepatic stellate cells. Our findings suggest a novel mechanism linking susceptibility to advanced fibrosis and subsequently indirectly to HCC, to the level of MICA expression through TGF-β1-dependent mechanisms.

## Introduction

Chronic hepatitis C virus (HCV) infection is a leading cause of cirrhosis, hepatocellular carcinoma (HCC) and liver transplantation, with an estimated 339,000 people dying annually from complications^1^. Advanced liver fibrosis or cirrhosis represents the major risk factor for developing liver-related complications and mortality, but currently there are no approved anti-fibrotic therapies^2^.

The rate of liver fibrosis progression varies greatly according to disease etiology and also between individuals; the latter is at least partially attributable to genetic factors. In chronic HCV infection, host genetics play a pivotal role in shaping the immune response, virus-host interactions and ultimately the predilection to and progress of liver fibrosis^3,4^. This risk is likely polygenic and dependent on multiple genetic and epigenetic factors since variations in single loci are usually of modest effect size and explain only a small fraction of the phenotype^5^. This has led in the recent past to a shift towards the discovery of novel variants with limited effects which ultimately could guide the development of polygenic scores with high predictive value.

Two genome wide association studies (GWAS) have investigated the risk of HCV-related HCC in Japanese patients. The first identified a locus in the 5′ flanking region of the MHC class I-related chain A (*MICA*) on 6p21.33 (rs2596542) to be strongly associated with HCC and the progression from CHC to HCC^6^. MICA is a ligand for natural-killer group 2 member D (NKG2D), a highly conserved C-type lectin-like membrane glycoprotein and one of the major activating receptors on NK cells^7^. The second GWAS identified a susceptibility locus near *DEPDC5* (rs1012068)^8^. The function of DEPDC5 is not well understood, but has been linked with hereditary forms of epilepsy^9^, bladder cancer^10^ and malignant glioblastomas^11^.

Complicating the interpretation of these findings however is that HCC development in chronic HCV infection is tightly linked to hepatic fibrosis with 90% of cases arising in cirrhotic livers^12^. Hence, risk variants that predispose to fibrosis could be associated with HCC formation without direct causality and disentangling the two is problematic. In this regard, little is known about the potential effect of variants in *MICA* and *DEPDC5* on liver fibrosis since both GWAS were done in patients in whom liver biopsy was not available. Importantly, functional data on the role of these variants with regard to both fibrosis pathways or HCC development are limited. The available literature is also restricted to Japanese populations with chronic HCV infection and HCC, while a single report in Caucasians^13^ suggests that *DEPDC5* but not *MICA* is associated with fibrosis progression.

Here we sought to dissect the role of *MICA* rs2596542 and *DEPDC5* rs1012068 to liver fibrosis and to HCV-related HCC. To do this, these variants were assessed in 1,501 patients with CHC of Caucasian ancestry in whom liver biopsy was available and were compared to 188 patients with CHC-related HCC. We undertook functional studies to explore the mechanisms that might underlie the genetic association with fibrosis.

## Results

### Patient characteristics

The clinical, demographic and biochemical characteristic of the patients in the cohort with chronic HCV infection (n = 1501) are presented in Supplementary Table 1. The genotype distribution of *MICA* rs2596542 and *DEPDC5* rs1012068 was in Hardy-Weinberg equilibrium and is presented in Supplementary Table 2. The minor allele frequency (MAF) for the two variants was similar to that observed in a healthy European population from the 1000 genome project (http://browser.1000genomes.org), which has some difference from the Japanese population.

### Association of *MICA* rs2596542 and *DEPDC5* rs1012068 with viral and clinical characteristics

To explore if baseline clinical variables differ between chronic HCV patients according to *MICA* rs2596542 or *DEPDC5* rs1012068 genotype, we examined the association of the genotypes with baseline clinical variables; the results are presented in Supplementary Tables 3 and 4, respectively. There was no evidence of association between either rs2596542 or rs1012068 genotype with any clinical variable (i.e. age, BMI, baseline levels of ALT, AST, GGT, ALP, platelets, leukocytes, HCV-RNA quantification, gender frequency or HCV genotype distribution).

### *MICA* rs2596542, but not *DEPDC5* rs1012068 is associated with fibrosis severity

**Figure 1:**
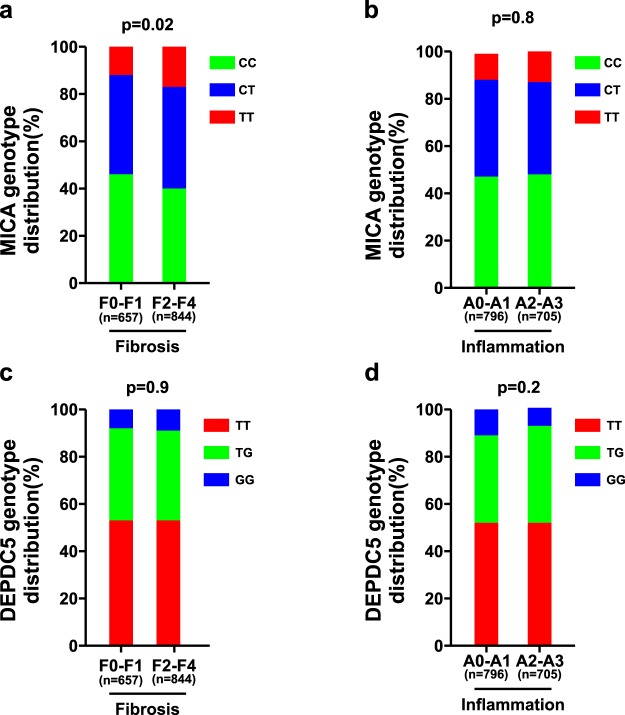
Association of *MICA* rs2596542 and *DEPDC5* rs1012068 with inflammation, fibrosis stage. Association of *MICA* rs2596542 with fibrosis stage (**a**) and inflammation (**b**) and *DEPDC5* rs1012068 with fibrosis stage (**c**) and inflammation (**d**) in the CHC cohort (n = 1501). P-values are univariate and provided for the additive model of inheritance. *MICA* rs2596542 (T) allele is the risk allele, the same risk allele in the GWAS by Kumar *et al*.^6^. *DEPDC5* rs1012068 (G) allele is the risk allele, the same risk allele in the GWAS by Miki *et al*.^8^.

We next assessed the association between *MICA* rs2596542 and *DEPDC5* rs1012068 and liver damage (hepatic inflammation and fibrosis). The distribution of rs2596542 and rs1012068 genotypes according to histological features is depicted in Fig. 1.

First, we examined the association of the two genetic variants with fibrosis. In multivariate analyses adopting an additive model adjusted for age, gender, BMI, HCV genotype and alcohol intake, *MICA* rs2596542 was significantly associated with stage of fibrosis (adjusted estimate, 0.072, SE, 0.051; p = 0.01) (Supplementary Table 5). In further analysis when the cohort was segregated into those with mild (F0–1) versus advanced fibrosis (F2–4), carriage of each copy of the *MICA* rs2596542 (T) allele was associated consistently with a significant increased risk of advanced fibrosis, independent of age, gender, BMI, HCV genotype and alcohol intake (OR: 1.47, 95% CI: 1.05–2.06, p = 0.02) (Supplementary Table 6). Similarly, rs2596542 was associated with severe fibrosis (F3-F4) (OR: 1.43, 95% CI: 1.03–1.99, p = 0.03). On the contrary, we did not observe any association between *DEPDC5* rs1012068 and stage of fibrosis (Supplementary Table 5); the distribution of rs1012068 genotypes was also not significantly different according to the presence or absence of significant or severe fibrosis (Supplementary Table 6).

We next examined the association of the two genetic variants with hepatic inflammation defined by liver histopathology scored according to METAVIR. Neither *MICA* rs2596542 nor *DEPDC5* rs1012068 demonstrated any association with inflammation grade (Supplementary Table 5). No association was also observed when the cohort was dichotomized into absent/mild (A0–A1) versus moderate/severe (A2–A3) inflammation (Supplementary Table 6). Consistently, no difference was noted in serum liver enzymes (ALT or AST; as indices of liver injury) according to rs2596542 or rs1012068 (Supplementary Tables 3 and 4, respectively).

### Association between *MICA* rs2596542 and fibrosis progression

To validate these observations, we undertook analysis in the 815 patients from the entire cohort with chronic HCV infection and a known duration of infection. This allowed assessment of the relationship to fibrosis progression without the inherent biases of cross sectional analyses. The baseline characteristics of the cohort were similar among subjects included and not included in the fibrosis progression sub-analysis (Supplementary Table 7). As the fibrosis progression rate (FPR) may not be constant over time^14^, we used Cox-proportional hazards analysis. In this analysis, rs2596542 was associated with an increased hazard of progression to significant fibrosis (≥F2) in a multivariate model that included age, gender, BMI and HCV genotype (hazards ratio: 1.41, 95% CI: 1.04–1.90, p = 0.02 per each T allele). In contrast, *DEPDC5* rs1012068 was not associated with FPR (hazards ratio: 1.06, 95% CI: 0.76–1.48, p = 0.7) (Supplementary Table 8). In sum, these data suggest that *MICA* rs2596542 but not *DEPDC5* rs1012068 is associated with fibrosis severity and fibrosis progression.

### Chronic HCV infection increases hepatic MICA expression

**Figure 2:**
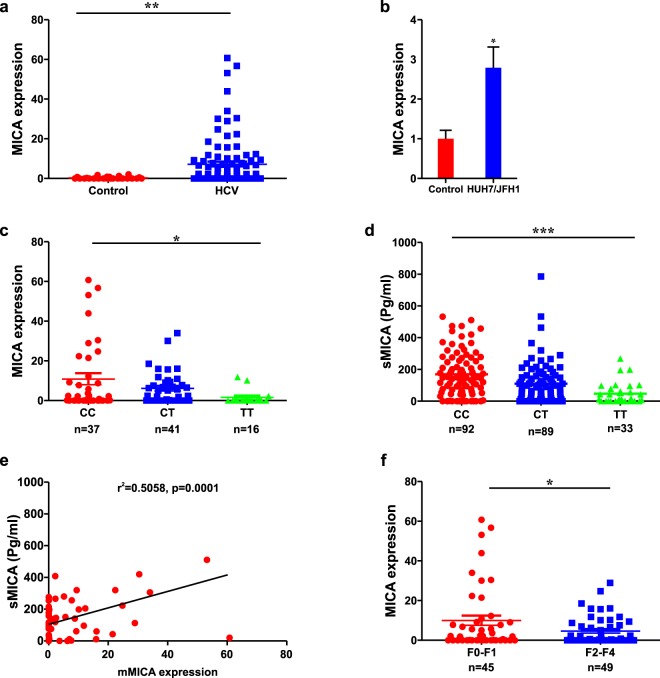
Hepatic and serum MICA expression. Relative hepatic MICA mRNA expression in 94 patients with chronic hepatitis C virus infection and 28 controls (**a**); relative MICA mRNA expression in Huh7 cells infected with the JFH1 strain hepatitis C virus as compared to mock infected control cells (**b**); correlation between *MICA* rs2596542 genotype and hepatic MICA mRNA (**c**) and soluble MICA levels (n = 214) (**d**). The x axis shows the genotypes at rs2596542 and the y axis shows MICA expression level relative to GAPDH by quantitative real-time PCR or the concentration of soluble MICA in pg/ml by ELISA, respectively. The level of hepatic MICA mRNA correlates positively with serum soluble MICA levels in a sub-cohort of CHC with available RNA and serum (n = 58) (**e**); hepatic MICA mRNA according to hepatic fibrosis (**f**). The x axis shows hepatic fibrosis dichotomized as absent/mild (METAVIR stage F0–F1) or moderate/severe (METAVIR stage F2–F4), and the y axis shows hepatic MICA expression. The number of independent samples tested in each group is shown below the figure and P value was calculated using the two-tailed Student’s t-test or ANOVA test with Tukey test for correction for multiple comparisons.

In the subsequent studies reported below, we sought to define the functional basis for the association of variants in *MICA* to liver fibrosis. The expression of MICA is low in healthy tissues but can be induced by cellular stressors such as viral infection^15^; the effect of HCV infection on hepatic MICA expression is not known. We therefore compared MICA expression in liver from patients with HCV and from control, non-infected subjects. RT-PCR demonstrated increased MICA mRNA expression in patients with chronic HCV infection (Fig. 2a). We then assessed MICA expression in the JFH1/Huh7 *in vitro* model of replicating HCV. Consistently, Huh7 cells infected with the JFH1 strain of HCV demonstrated a 3-fold upregulation of MICA mRNA compared to control uninfected cells (Fig. 2b), p < 0.05).

The biological effect of MICA in HCV-infected liver likely reflects a complex interaction between the different cell types within an inflamed liver that ultimately leads to fibrosis. Hence, we examined the expression of MICA in four primary human hepatic cell types, namely hepatocytes, Kupffer cells, hepatic sinusoidal endothelial cells and stellate cells by real time PCR. The highest expression of MICA was in stellate cells followed by Kupffer cells, while sinusoidal cells and hepatocytes had low expression (Supplementary Fig. 1).

### Membranous and soluble MICA levels associate with MICA genotype and fibrosis severity

Whether the *MICA* risk allele is associated with altered membrane bound (mMICA) expression in liver tissue from patients with chronic HCV infection is not known as all available current data are based on the soluble form (sMICA) present in serum^6,16^. As shown in Fig. 2c, we observed a significant association between rs2596542 genotype and MICA mRNA expression; the (T) allele associated with lower hepatic MICA mRNA in the 94 patients with available liver tissue (P = 0.01, p = 0.02 after adjustment for age and sex).

We then measured soluble MICA (sMICA) by ELISA in serum samples from a sub-cohort of 214 chronic HCV infection patients; their characteristics are summarized in Supplementary Table 9 and matched the overall cohort. Similar to mMICA, compared to patients harboring the *MICA* rs2596542 CC genotype, sMICA levels were significantly reduced in those with the risk T allele (Fig. 2d). We determined the correlation of mMICA to sMICA in 58 patients in whom paired liver and serum data was available. In this analysis, levels of sMICA associated with the expression of mMICA (p = 0.0001) (Fig. 2e).

In total, MICA expression is upregulated in HCV infection in an rs2596542 genotype dependent manner with individuals carrying rs2596542 (T) expressing lower levels of mMICA and sMICA. This suggests that differences in expression levels of mMICA according to genotype are not a consequence of differing shedding rates of MICA.

### MICA expression and liver fibrosis

The overall contribution of the MICA/NKG2D pathway to fibrosis is unclear. Interestingly and consistent with the genetic data, hepatic MICA expression was significantly lower in subjects with significant hepatic fibrosis (F2–F4) compared to those with no or mild hepatic fibrosis (F0/1) (n = 94) (Fig. 2f). MICA expression did not correlate with hepatic inflammation, liver enzymes, HCV-RNA levels and genotype, age or gender. Similarly, sMICA levels were decreased in patients with significant fibrosis compared to those without, though this was not significant (Supplementary Fig. 2).

### MICA expression is regulated by TGF-β1 in hepatic stellate cells

We next thought to explore how differential expression of hepatic MICA could be linked to fibrosis progression. Hepatic stellate cells (HSC) are responsible for the deposition of extracellular matrix that is evident histologically as fibrosis^17^ and as we have shown, also have the highest expression of MICA. Killing of activated HSCs by NK cells to ameliorate liver fibrosis is in part by a MICA/NKG2D-dependent manner^18^. Thus, we investigated the regulation of MICA expression on HSCs in a fibrotic context.

**Figure 3:**
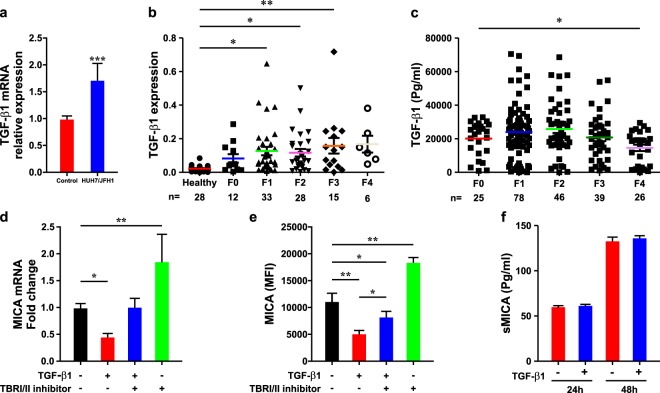
Transforming growth factor beta influences MICA expression. Relative TGF-β1 mRNA expression in Huh7 cells infected with the JFH1 strain of the hepatitis C virus compared to mock infected control cells (**a**); relative hepatic TGF-β1 mRNA expression in 94 patients with chronic hepatitis C virus infection and 28 controls according to fibrosis stage (**b**); serum concentration of TGF-β1 in pg/ml levels according to hepatic fibrosis (n = 214) (**c**); MICA mRNA expression and protein levels at the hepatic stellate cell surface modulation by TGF-β1. MICA mRNA expression was assessed in control, TGF-β1 (2 ng/ml) with or without LY2109761 (a TGFβR-I/II inhibitor) (100 nM, was added 90 minutes before TGF-β1), or LY2109761 (100 nM) treated cells for 24 hours by real-time PCR (**d**), or 48 hours for flow cytometry (**e**); the level of soluble MICA was assessed in the supernatant of control or TGF-β1 (2 ng/ml) treated LX2 cells for 24 and 48 hours by ELISA (**f**). Results are expressed, as mean ± sem (n = 3) and P value was calculated using the two-tailed Student’s t-test or ANOVA test with Tukey test for correction for multiple comparisons. *P < 0.05,**P < 0.001, ***P < 0.0001.

Surface expression of MICA has been reported to be down-regulated by TGF-β1^19^, an established growth factor mediating liver injury and fibrosis^12^. Hence, we characterized TGF-β1 mediated regulation of MICA expression on HSCs. We first assessed expression of TGF-β1 mRNA after HCV infection *in vitro* using qRT-PCR. This revealed a significant increase in TGF-β1 in HCV-infected JFH-1/Huh7 cells compared to non-infected cells (Fig. 3a). Consistently, we observed increased TGF-β1 transcription in liver from chronic HCV infected liver samples compared to healthy controls (Fig. 3b). Within the chronic HCV infection group, the expression of TGF-β1 mRNA was significantly up-regulated with advancing of fibrosis stage (Fig. 3b) and this was consistently observed in the sub-cohort with available serum described above (n = 214) (Fig. 3c).

Given these results, we examined for the effect of TGF-β1 on MICA expression on a human HSC-derived cell line (LX2). We observed a marked repression of MICA mRNA transcripts in TGF-β1 stimulated LX2 cells (Fig. 3d), as well as reduced surface expression of MICA, by flow cytometry (Fig. 3e). For confirmation that this effect was mediated through the TGFβR, we used a specific pharmacological inhibitor of TGFβR-I/II (using LY2109761) and this as expected, reversed the inhibitory effect of exogenous TGFβ1 on MICA expression (Fig. 3d and e). To characterize the role of endogenous HSC-derived TGF-β1 in the regulation of MICA expression, we used LX2 cells treated with LY2109761 and demonstrated a significant induction of MICA mRNA transcripts and surface expression levels (Fig. 3d and e).

Lastly, previous reports have observed a positive effect of TGF-β1 treatment on the expression of some proteases including MMP-9. Since MICA surface expression can be modulated at a post-translational level by proteolytic cleavage^20^, we asked whether shedding of sMICA was enhanced in TGF-β1 stimulated cells, thus contributing to the attenuation of surface expression on these cells. To this end, we measured sMICA in cell culture supernatant and showed that the level of sMICA released by TGF-β1 stimulated cells was not different compared with control cells suggesting that cleavage of MICA is not mediated by TGF-β1-dependent proteases (Fig. 3f).

### MICA polymorphism is not associated with HCC

 As *MICA* was originally discovered as a risk locus for HCC in Japanese patients, we investigated the role of *MICA* in hepatocarcinogenesis. We compared *MICA* genotype distribution in patients with chronic HCV-HCC (n = 188) to non-HCC from the entire chronic HCV infection cohort described above (n = 1,501; Supplementary Table 10). We observed no difference in *MICA* rs2596542 distribution between subjects with HCC and those without. Because HCC mainly occurs in the context of cirrhosis, we undertook similar analysis restricting the comparison to those with HCC versus those with chronic HCV infection and cirrhosis (n = 210). Again, no difference in *MICA* rs2596542 distribution was observed between subjects with HCC and those with cirrhosis (p = 0.77). Results were not changed after adjustment for age and sex. These findings imply that *MICA* rs2596542 polymorphism is unlikely to be directly associated with the occurrence of HCC.

## Discussion

**Figure 4:**
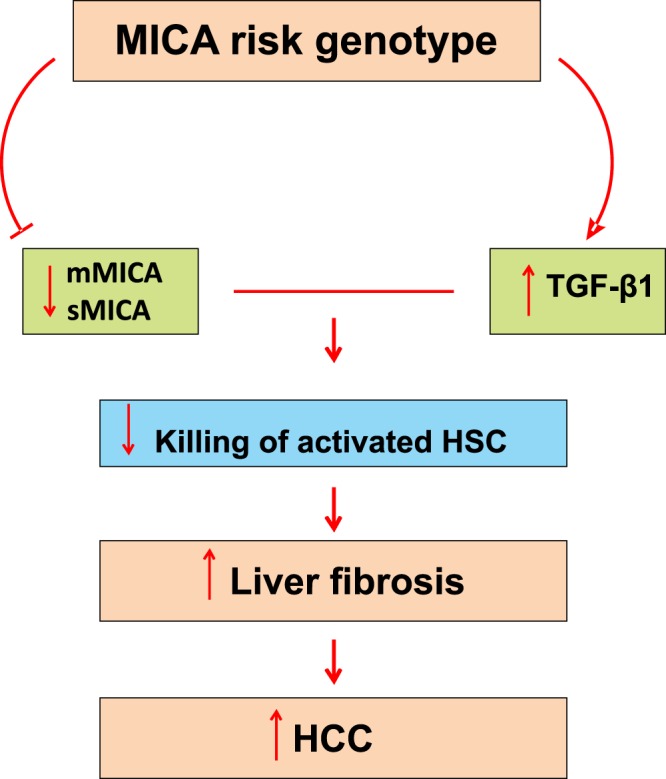
Proposed model for the effect of *MICA* rs2596542 in modulating liver fibrosis risk. Patients carrying the *MICA* risk genotype have low hepatic and serum MICA expression. TGF-β1 further suppresses MICA expression on hepatic stellate cells, leading to increased liver fibrosis.

Here, we report that a genetic variant in *MICA* originally identified by GWAS as a risk variant for susceptibility to HCV-associated HCC in Japanese patients^6^ associates with fibrosis severity and progression, but not with HCC. Functionally our data suggest that the association with HCC is plausibly mediated by indirect effects through modulation of fibrosis risk. Following the data for effects on fibrosis progression, we show *MICA* genotype-dependent reductions in hepatic MICA gene expression and soluble MICA protein levels. Tying these findings to fibrosis, TGF-β1 was increased with advancement of fibrosis and there was a corresponding reduction in MICA expression. *In vitro*, TGF-β1 treatment resulted in MICA transcriptional and surface protein repression on HSC cells. These effects we propose leads to an increased likelihood of fibrosis in those with the risk genotype. Our findings therefore suggest a molecular explanation for the association of rs2596542 with liver fibrosis (Fig. 4).

Consistent with the present findings in the setting of chronic HCV infection, a recent report suggests that *MICA* polymorphisms (MICA*012:01/02, MICA*017 and MICA*027) are associated with liver fibrosis in schistosomiasis^21^. It is noteworthy that in the original GWAS report of *MICA* in HCV-related HCC, Kumar *et al*., did not use HCV-related cirrhosis without HCC as their control cohort, masking any potential confounding by fibrosis^6^. This and the failure to find an association with *MICA* in a similar Japanese study published 2 months later^8^, as well as the functional data we provide, suggest that the reported association with HCC actually relates to liver fibrosis risk.

MICA is a stress inducible protein present at very low levels in healthy tissues, but can be induced by cellular stresses such as viral infection^15^. Consistently, we demonstrated increases in MICA expression during the early stages of HCV infection, while it decreases with increasing fibrosis in an rs2596542 genotype dependent manner. Furthermore, the (T) risk allele correlated with lower mMICA in liver. Consistent with other groups^6,16^, we demonstrated decreased expression levels of sMICA in the serum of patients with the (T) rs2596542 allele.

The mechanisms that link *MICA* genotypes with fibrosis risk have not been previously elucidated. MICA is a ligand for the activating NK cell receptor NKG2D and this interaction shapes the anti-fibrotic capacity of NK-cells via elimination of activated HSCs^18,22^ We verified that HCV infection is associated with an increase in TGF-β1 expression in liver^23^ and the latter induces marked repression of MICA expression in HSC. This suggests that *MICA* genotype might be associated with alterations in TGF-β1 (through as yet undefined mechanisms) and may underlie the accelerated fibrosis we observed.

We did not observe any association between *DEPDC5* rs1012068, the other variant identified by GWAS as a risk variant for HCC^8^, with either fibrosis or HCC. A large Japanese study of 2,266 HCV patients who had eradicated HCV and with long term follow up to 20 years, was also unable to find any association between *DEPDC5* SNPs at rs1012068 and the cumulative risk of HCC^24^. Some studies have reported an association of *DEPDC5* with fibrosis but with opposite effects of the risk allele^13,25,26^. A recent smaller study conducted in Europeans suggested an association between *DEPDC5* rs1012068 but not *MICA* rs2596542 and risk of cirrhosis in chronic HCV infection^13^. The reasons for the discrepancy between our study and this work are unclear, but may relate to differences in baseline characteristics and that the association analyses for fibrosis were performed differently, as those authors compared F0-F1 vs. F4. Interestingly, in that study, *MICA* rs2596542 showed a tendency for significance with cirrhosis (p = 0.07) in the discovery cohort (n = 477) but was not investigated in the validation cohort. The function of DEPDC5 in liver needs to be defined.

Our study has some limitations, including the fact that the sample size of the PCR and ELISA sub-cohorts was relatively modest. In addition, whether *MICA* rs2596542 is the functional polymorphism or if there are other variants in linkage disequilibrium and the detailed functional mechanisms for the effects are still not fully understood.

In conclusion, we have demonstrated that *MICA* rs2596542 is associated with liver fibrosis progression, likely mediated and amplified in part through TGFβ-1 dependent mechanisms.

## Methods

### Patient cohort

The study comprised 1689 consecutive patients (1,501 with CHC and 188 with HCV-related HCC) from the International Liver Disease Genetics Consortium (ILDGC) database. Details of the cohort and inclusion criteria have been reported^27–29^. Briefly, all subjects who had a liver biopsy with scoring for fibrosis stage and disease activity before anti-viral treatment, and genomic DNA were included. For those with CHC, those with HCV RNA serum positivity of Self-reported Caucasian descent were included. Patients were excluded if they had evidence of co-infection with other hepatitis viruses, other liver diseases by standard tests or current or previous hepatic decompensation. Diagnosis of HCC was based on the EASL–EORTC Clinical Practice Guidelines^30^.

The study protocol was conformed to the ethical guidelines of the 1975 Declaration of Helsinki. Ethics approval was obtained from the Human Research Ethics Committees of the Western Sydney Local Health District and the University of Sydney. All other sites had ethics approval from their respective ethics committees and study was performed according to the recommendations of the centers involved. Written informed consent for genetic testing was obtained from all participants.

### Clinical and laboratory assessment

The following data were collected at time of liver biopsy from all patients: gender, age, ethnicity, recruitment center, alcohol intake (g/day), body mass index (BMI) and routine laboratory tests. BMI was calculated as weight divided by the square of the height (kg/m^2^).

### Methods to estimate the duration of infection

Fibrosis progression was examined in 815 chronic HCV infection patients with a reliable estimated duration of infection, as previously reported^31,32^. Briefly, for subjects with a history of injecting drug use, the time of infection was estimated using the reported “first year of injection”. For patients with a history of blood transfusion, the onset of infection was assumed to be the year of transfusion. For patients with a history of occupational exposure, the onset of infection was assumed to be the first year of needle stick exposure. The duration of infection was calculated by subtracting the estimated age at infection from age at biopsy.

### Genotyping

Genotyping for *MICA* rs2596542 and *DEPDC5* rs1012068 was contracted to the Australian Genome Research Facility (AGRF; QLD, Australia). 1051 samples were genotyped using the Sequenom MassARRAY system and iPLEX Gold chemistry while 638 samples were genotyped using the TaqMan SNP genotyping allelic discrimination method (Applied Biosystems, Foster City, CA, USA). All genotyping was blinded to clinical variables.

### Liver histopathology

Liver histopathology was scored according to METAVIR^33^. Fibrosis was staged from F0 (no fibrosis) to F4 (cirrhosis). Necroinflammation (A) was graded as A0 (absent), A1 (mild), A2 (moderate) or A3 (severe). The inter-observer agreement between pathologists was studied previously and was good (κ = 77.5) for METAVIR staging using κ statistics^34^.

### Other methods

Methods for cell culture, real-time PCR, ELISA and flow Cytometry are described in Materials and Methods of the Supporting Information

### Statistical analysis

Statistical analyses were performed using the statistical software package SPSS for Windows, version 21 (SPSS, Chicago, IL). All tests were two-tailed and p values < 0.05 were considered significant. Details of statistical analyses are described in the Materials and Methods of the Supporting Information.

## Electronic supplementary material

Supplementary file

## Data Availability

All data are provided within the main text and Supplementary File.
