# Supplementary material for: A variant in the MICA gene is associated with liver fibrosis progression in chronic hepatitis C through TGF-β1 dependent mechanisms
Source: Sci Rep. 2019 Feb 5;9:1439. doi: 10.1038/s41598-018-35736-2 (PMC6363805; doi:10.1038/s41598-018-35736-2)
Supplement: Supplementary file 1 — Supplementary file [file 41598_2018_35736_MOESM1_ESM.docx]

**A variant in the MICA gene is associated with liver fibrosis progression in chronic hepatitis C through TGF-β1 dependent mechanisms**

Rasha El Sharkawy, Ali Bayoumi, Mayada Metwally, Alessandra Mangia, Thomas Berg, Manuel Romero-Gomez, Maria Lorena Abate,William L. Irving, David Sheridan, Gregory J. Dore, Ulrich Spengler, Pietro Lampertico, Elisabetta Bugianesi, Martin Weltman, Lindsay Mollison, Wendy Cheng, Stephen Riordan, Rosanna Santoro, Rocío Gallego-Durán, Janett Fischer, Jacob Nattermann, RobertaD’Ambrosio, Duncan McLeod, Elizabeth Powell, Olivier latchoumanin, Khaled Thabet, Mustafa A.M. Najim, Mark W. Douglas, Christopher Liddle, LiangQiao, Jacob George and Mohammed Eslam, for the International Liver Disease Genetics Consortium (ILDGC).

**Supplementary methods:**

**Reagents**

transforming Growth Factor Beta 1 (TGF-β1) were from R&D systems, Dulbecco's Modified Eagle Medium (DMEM; Invitrogen, Karlsruhe, Germany) and Fetal Bovine Serum (FBS) (Invitrogen, Karlsruhe, Germany), LY2109761 (Cayman Chemical, Michigan, USA), RNeasy kit (Qiagen), qscript (Quanta Biosciences, Gaithersburg, MD, USA), Trypsin (Sigma Aldrich, Germany).

**Cell lines, Cell culture and HCV Transfection**

Huh7 cells were maintained in Dulbecco's modified eagle medium supplemented with 10% fetal bovine serum, L-glutamine, non-essential amino acids, penicillin and streptomycin (Invitrogen). The cells were transfected with the JFH1 strain of hepatitis C virus and passaged in culture for 3 weeks until over 90% of cells were infected. HCV infection was confirmed by immunofluorescence using specific antibodies against HCV NS5A protein. The HSC line LX2 (a kind gift of Professor S. Friedman, Mount Sinai School of Medicine, New York, New York, USA) was maintained in DMEM containing 2% FCS. LX2 cells were treated with 2 ng/ml human recombinant transforming growth factor beta 1 (TGF-β1) (R&D systems,, Abingdon, UK) or mock-treated with vehicle, in some conditions an inhibitor of TGFβR-I/II (using LY2109761, 100 nM) was added 90 minutes before TGF-β1 and cells were harvested after 24 hours for the mRNA experiments or 48 hours for the Flow Cytometry experiments. In another experimental condition, cells were treated only with and without LY2109761 for the same duration.

**RNA extraction and cDNA synthesis**

RNA was extracted using the RNeasy kit according to the manufacturer’s instructions. RNA quality and concentration was assessed using the Agilent 2100 Bioanalyser (Agilent, Waldbronn, Germany). cDNA was prepared using qscript in a Mastercycler gradient 5331 (Eppendorf AG, Hamburg, Germany).

**Quantitative real time reverse transcription PCR.**

mRNA expression levels were determined using specific primer and master mix according to the manufacturer’s protocol. GAPDH was used as the house keeping gene. Expression was measured using CT values, and the ΔΔCt method was used to calculate relative mRNA expression levels normalized to those of GAPDH. MICA mRNA expression was also assessed in human primary hepatocytes, human primary hepatic stellate cells, human primary hepatic sinusoidal endothelial cells (ScienceCell) and human primary Kupffer cells (Thermofisher).

**Flow Cytometry.**

Cultured cells were detached with Trypsin (Sigma Aldrich, Germany), then non-permeabilized cells were incubated with monoclonal antibodies against MICA/B (Abcam, UK), washed, and incubated with a fluorescein isothiocyanate– labeled goat antimouse secondary antibody. Cells were suspended in 0.5 mL of PBS stained with propidium iodide and analyzed on a FACScan flow cytometer from Beckman Coulter (Fullerton, CA). Staining with the isotype control was run in parallel.

**ELISA**

Soluble MICA and active TGF-β1 measurements were performed using an ELISA kit (R&D), according to the manufacturer’s instructions.

**Statistical Analysis**

Statistical analyses were performed using the statistical software package SPSS for Windows, version 21 (SPSS, Chicago, IL). All tests were two-tailed and p values <0.05 were considered significant. Results are expressed as mean ± SD (standard deviation), median and range or number (percentage) of patients. The Student’s *t*-test or non-parametric, i.e. Wilcoxon-Mann-Whitney U-test or Kruskal-Wallis tests were used to compare quantitative data, as appropriate. χ^2^ test and Fisher-exact tests were used for comparison of frequency data and to evaluate the relationships between groups. All tests were two-tailed and p values <0.05 were considered significant. For analysis, the *MICA* rs2596542 and *DEPDC5* rs1012068 variants were coded in an additive model. The additive model was tested using the Cochran-Armitage test for trend. Power calculations were performed and assuming rs2596542 MAF of 0.40 and rs1012068 MAF of 0.27. The consistency of genotype frequencies with Hardy-Weinberg equilibrium (HWE) was tested using an exact test. Multiple logistic regression models were fitted to binary traits; necroinflammation was dichotomized as absent/mild (METAVIR score A0-A1) or moderate/severe (Metavir score A2-A3), and fibrosis as absent/mild (METAVIR score F0-1) or significant (Metavir score F2-4), no/moderate fibrosis (F0-F2) or severe fibrosis (F3-F4). The odds ratio estimates the relative change in the rate of the outcome (e.g., significant fibrosis) per unit increase in the explanatory variable. In CHC, multivariate logistic regression analysis was adjusted for biologically relevant covariates associated with the risk of liver disease progression (age, gender, BMI, alcohol consumption and HCV-genotype). In HCC, the model was adjusted for known risk factors, including age, gender, BMI and Child-Pugh score. HCV-RNA levels were log-transformed before entry into the model. Results are expressed as odds ratios and 95% CI. For fibrosis progression rate (FPR), we used Cox regression analysis to model the time taken for significant fibrosis (≥ F2) to develop. For this, after checking the normality of the quantified variables, appropriate logarithmic transformations were made. We considered estimated age at infection as the starting point and the first liver biopsy showing significant fibrosis (failure time) or the last liver biopsy showing an absence of significant fibrosis in the absence of treatment (censored time) as the end point. A Cox proportional-hazards regression model was fitted, and the covariates were considered significant if p <0.05. Multivariate adjusted analyses were used with age, gender, BMI, alcohol consumption and HCV-genotype, as covariates.

**Supplementary results:**

**
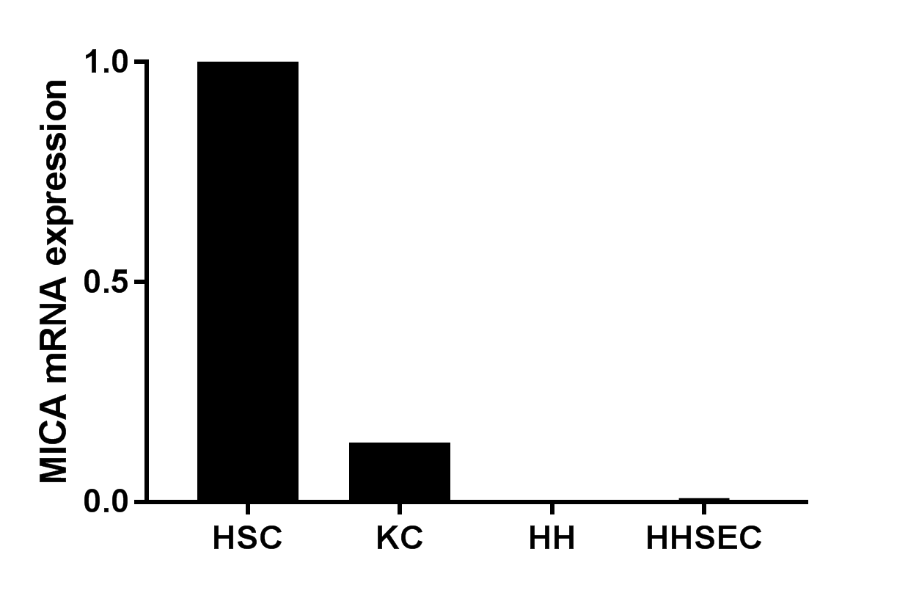
**

**Supplementary Figure 1: MICA mRNA expression in human primary hepatic cell types.** Gene expression level was assessed by quantitative polymerase chain reaction. The tissue or the cell line with the highest expression was assigned a value of 1. HH, human hepatocytes; HHSEC, human hepatic sinusoidal endothelial cell; HSC, hepatic stellate cell; KC, Kupffer cell.

**Supplementary figure 2: sMICA according to hepatic fibrosis in CHC patients (n=214).** The x axis shows hepatic fibrosis dichotomized as absent/mild (METAVIR stage F0–F1) or moderate/severe (METAVIR stage F2–F4), and the y axis shows the concentration of soluble MICA in pg/ml. The number of independent samples tested in each group is shown below the figure and the P value was calculated using the two-tailed Student's t-test and was not significant.

**Supplementary Table 1: Demographic and clinical characteristics of the cohort of patient with CHC (n=1501).**

| **Variables** |  |
| --- | --- |
| **Age (Years)** | 44 (38.4-51) |
| **Male (%)** | 977 (65.1) |
| **Body Mass Index (Kg/m^2^)** | 26.3 (23.6-29.7) |
| **ALT (IU/L)** | 74 (48-124) |
| **AST (IU/L)** | 65 (38.4-51) |
| **GGT (IU/L)** | 56 (29-100) |
| **Platelet (10^9^/L)** | 211 (166-253) |
| **HCV-RNA log_10_** | 5.92 (5.5-6.3) |
| **Leukocyte (10^9^/L)** | 6.8 (5.6-8.5) |
| **HCV-genotype (%) 1, 2, 3, 4** | 1028 (68.5), 134 (8.9), 310 (20.7), 20 (1.9) |
| **Liver fibrosis (%)** |  |
| F0-F1 | 657 (43.8) |
| F2-F4 | 844 (56.2) |
| **Inflammation score (%)** |  |
| A0-A1 | 796 (53) |
| A2-A3 | 705 (47) |

*Values are median (interquartile range), or number (%).*

**Supplementary Table 2. Distribution of *MICA* rs2596542 and *DEPDC5* rs1012068 genotypes and Hardy-Weinberg equilibrium.**

|  |  | **1000 genome** | |
| --- | --- | --- | --- |
| ***Variant*** | **CHC (Caucasian population) (n=1501)** | **EUR** | **JPT** |
| ***MICA* rs2596542** |  |  |  |
| CC | 535 (35.6) |  |  |
| CT | 697 (46.4) |  |  |
| TT | 269 (18) |  |  |
| C allele | 59% | 60% | 67% |
| T allele | 41% | 40% | 33% |
| ***DEPDC5* rs1012068** |  |  |  |
| TT | 791 (52.7) |  |  |
| TG | 580 (38.6) |  |  |
| GG | 130 (8.7) |  |  |
| T allele | 72% | 73% | 88% |
| G allele | 28% | 27% | 12% |

*p =0.1 for both variants. P values were calculated by chi square test, p >0.05 indicates no deviation from Hardy-Weinberg equilibrium. The allele frequencies for the two variants in a healthy European and Japanese population from the 1000 genome project (*[*http://browser.1000genomes.org)are*](http://browser.1000genomes.org)are) *presented.*

**Supplementary Table 3. Characteristics of 1501 patients with CHC according to *MICA* rs2596542 genotype**

| **Variable** | ***MICA rs2596542 Genotype*** | | | ***P*-value** |
| --- | --- | --- | --- | --- |
|  | **CC (n=535)** | **CT (n=697)** | **TT (n=269)** |  |
| **Age at time of biopsy (years)** | 44 (39-50) | 44.2 (38-51) | 44.6 (38-52) | 0.8 |
| **Male Gender (%)** | 361 (67.5) | 439 (63) | 177 (65.8) | 0.2 |
| **HCV genotype 3 (%)** | 104 (19.4) | 144 (20.7) | 62 (23) | 0.4 |
| **HCV-RNA log_10_ *** | 5.92 (5.59-6.17) | 5.91 (5.43-6.28) | 5.92 (5.7-6.4) | 0.1 |
| **Body Mass Index (Kg/m^2^)** | 26.6 (23.4-29.5) | 26.3 (23.8-30) | 26.6 (24.2-30.3) | 0.7 |
| **ALT (IU/L)** | 72 (46-119) | 74 (49-126) | 77 (49.7-139) | 0.4 |
| **AST (IU/L)** | 54 (37-85.7) | 54 (38-91) | 65 (44-99) | 0.06 |
| **GGT (IU/L)** | 57 (29-99) | 53 (27.7-97.2) | 62 (37-117) | 0.09 |
| **Alkaline phosphatase (IU/L)** | 74 (63-94) | 74 (62-93) | 79 (61-99) | 0.6 |
| **Platelet (x10^9^/L)** | 216 (171-259) | 231 (167.7-250) | 202 (150-246) | 0.1 |
| **Leukocyte (x10^9^/L)** | 7 (5.55-8.65) | 7.15 (5.9-8.7) | 6.25 (5.4-8.5) | 0.1 |

*Values are median (interquartile range), or number (%)*. **HCV-RNA viral load levels were available for 1221 patients. P-value was estimated using Kruskal-Wallis tests or Fisher-exact tests.*

**Supplementary Table 4. Characteristics of 1501 patients with CHC according to *DEPDC5* rs1012068 genotype***

| **Variable** | ***DEPDC5* rs1012068 *Genotype*** | | | ***P*-value** |
| --- | --- | --- | --- | --- |
|  | **GG (n=791)** | **GT (n=580)** | **TT (n=130)** |  |
| **Age at time of biopsy (years)** | 44 (38-51) | 45 (39-51) | 43 (38-48.4) | 0.2 |
| **Male Gender (%)** | 508 (64.2) | 382 (65.9) | 87 (66.9) | 0.7 |
| **HCV genotype 3 (%)** | 158 (20) | 122 (21) | 30 (23) | 0.6 |
| **HCV-RNA log_10_** | 5.92 (5.55-6.29) | 5.92 (5.55-6.23) | 5.92 (5.5-6.41) | 0.8 |
| **Body Mass Index (Kg/m^2^)** | 26.5 (23.9-29.5) | 26.4 (23.3-29.8) | 26.2 (23.1-30.1) | 0.9 |
| **ALT (IU/L)** | 72 (47-118) | 79 (50-137) | 71 (45-131) | 0.4 |
| **AST (IU/L)** | 55 (38-87) | 56 (38-92) | 56 (40-87) | 0.7 |
| **GGT (IU/L)** | 56 (31-99) | 56 (27-101) | 61 (32-100) | 0.9 |
| **Alkaline phosphatase (IU/L)** | 75 (63-96) | 73 (59-90) | 75 (64-94) | 0.1 |
| **Platelet (x10^9^/L)** | 210 (165-249) | 213 (169-253) | 224 (157-278) | 0.4 |
| **Leukocyte (x10^9^/L)** | 6.7 (5.62-8.3) | 6.65 (5.4-8.52) | 7 (6-9.2) | 0.07 |

*Values are median (interquartile range), or number (%)*. **HCV-RNA viral load levels were available for 1221 patients. P-value was estimated using Kruskal-Wallis tests or Fisher-exact tests.*

**Supplementary Table 5: Independent predictors of the degree of inflammation (A0–3) and stage of fibrosis (F0–4) in 1501 CHC patients.**

|  | **Degree of inflammation (A0–3)** | | | **Stage of fibrosis (F0–4)** | | |
| --- | --- | --- | --- | --- | --- | --- |
|  | **Estimate** | **SE** | ***P* value** | **Estimate** | **SE** | ***P* value** |
| **Age, years** | 0.226 | 0.004 | 0.0001 | 0.280 | 0.003 | 0.0001 |
| **Gender, female** | -0.078 | 0.043 | 0.01 | -0.097 | 0.065 | 0.0001 |
| **HCV genotype 3** | 0.030 | 0.0001 | 0.1 | 0.042 | 0.038 | 0.1 |
| **Alcohol (≥ 50 g/daily)** | 0.033 | 0.014 | 0.1 | 0.052 | 0.046 | 0.09 |
| **Body Mass Index (Kg/m^2^)** | 0.125 | 0.006 | 0.002 | 0.894 | 0.009 | 0.001 |
| ***MICA* rs2596542** | 0.058 | 0.138 | 0.1 | 0.072 | 0.051 | 0.01 |
| ***DEPDC5* rs1012068** | 0.018 | 0.002 | 0.6 | 0.015 | 0.01 | 0.4 |

*Multiple linear regression models were used to test the association of demographic, clinical and genetic factors with liver damage.* *Genetic analyses were calculated using an additive model with MICA rs2596542 (T) allele the risk allele, the same risk allele in the GWAS by Kumar et al (6). DEPDC5 rs1012068 (G) allele is the risk allele, the same risk allele in the GWAS by Miki et al (8).*

**Supplementary Table 6: Independent predictors of moderate/severe inflammation (≥A2) and significant fibrosis (≥F2) by logistic regression analysis in the chronic hepatitis C virus infection patient cohort (n=1501).**

|  | **Moderate/severe inflammation (≥A2)** | | | **Significant fibrosis (F2-F4)** | | |
| --- | --- | --- | --- | --- | --- | --- |
|  | **OR** | **95% CI** | ***P* value** | **OR** | **95% CI** | ***P* value** |
| **Age, years** | 1.029 | 1.020-1.038 | 0.0001 | 1.05 | 1.03-1.07 | 0.0001 |
| **Gender, female** | 0.731 | 0.609-0.879 | 0.001 | 0.65 | 0.51-0.83 | 0.001 |
| **HCV genotype 3** | 1.093 | 0.873-1.368 | 0.4 | 1.42 | 1.01-1.99 | 0.04 |
| **Body Mass Index (Kg/m^2^)** | 1.042 | 1.008-1.076 | 0.01 | 1.045 | 1.01-1.08 | 0.01 |
| **Alcohol (≥ 50 g/daily)** | 1.21 | 0.64-2.29 | 0.5 | 1.13 | 0.85-1.49 | 0.3 |
| ***MICA rs2596542*** | 1.18 | 0.67-1.98 | 0.6 | 1.47 | 1.05-2.06 | 0.02 |
| ***DEPDC5 rs1012068*** | 1.01 | 0.79-1.28 | 0.9 | 1.02 | 0.61-1.7 | 0.9 |

*Multiple logistic regression models were used to test the association of demographic, clinical and genetic factors with liver damage. Genetic analyses were calculated by using an additive model. The reference group was defined by mild inflammation (A0-A1), and fibrosis (F0−F1). MICA rs2596542 (T) allele is the risk allele, the same risk allele in the GWAS by Kumar et al (6). DEPDC5 rs1012068 (G) allele is the risk allele, the same risk allele in the GWAS by Miki et al (8).*

**Supplementary Table 7: Demographic and clinical characteristics of the sub-cohort (n = 815) of patients with CHC and a known estimated duration of infection.**

| **Variables** | **FPR* sub-cohort**  **(n=815)** |
| --- | --- |
| **Age** | 44 (38-50) |
| **Male (%)** | 548 (67.2) |
| **Body Mass Index (Kg/m^2^)** | 26.3 (23.4-29.7) |
| **ALT (IU/L)** | 81 (50-147) |
| **AST (IU/L)** | 60 (39-100) |
| **GGT (IU/L)** | 58 (31-104) |
| **Platelet (x10^9^/L)** | 226 (187-271) |
| **HCV-RNA log_10_** | 5.91 (5.55-5.92) |
| **HCV-genotype 3 (%)** | 204 (25) |
| **Liver fibrosis (%)** |  |
| **None/mild** | 382 (46.9) |
| **Moderate/severe** | 433 (53.1) |
| **Inflammation score (%)** |  |
| **None/mild** | 417 (51.2) |
| **Moderate/severe** | 398 (48.8) |

*Values are median (interquartile range), or number (%)*. *Fibrosis progression rate (FPR) was calculated by taking the ratio between the fibrosis stage and the estimated disease duration (in years).*

**Supplementary Table 8: Independent predictors for the hazard of significant fibrosis according to a Cox regression analysis in the sub-cohort (n = 815) of patients with CHC and a known estimated duration of infection.**

|  | **HR** | **95% CI** | **P-value** |
| --- | --- | --- | --- |
| **Age at infection, years** | 1.077 | 1.058-1.097 | 0.0001 |
| **Gender, female** | 0.823 | 0.681-0.994 | 0.043 |
| **HCV genotype 3** | 1.335 | 1.031-1.728 | 0.029 |
| **BMI, Kg/m^2^** | 1.010 | 0.985-1.036 | 0.4 |
| ***MICA rs2596542*** | 1.41 | 1.04-1.90 | 0.02 |
| ***DEPDC5 rs1012068*** | 1.06 | 0.76-1.48 | 0.7 |

*HR: the hazard; 95% confidence interval for the effect; hepatitis C virus (HCV) non-genotype 3 was considered the reference cohort.* *MICA rs2596542 (T) allele is the risk allele and DEPDC5 rs1012068 (G) allele is the risk allele.*

**Supplementary Table 9: Demographics and clinical characteristics of the hepatitis C patients and healthy controls of the ELISA sub-cohort.**

| **Variables** | **HCV (n=214)** |
| --- | --- |
| **Age (Years)** | 47.7 (40.6-53.8) |
| **Male (%)** | 141 (65.8) |
| **Body Mass Index (Kg/m^2^)** | 27.45 (24.3-21.3) |
| **ALT (IU/L)** | 81.5 (47-125) |
| **AST (IU/L)** | 67 (46-110) |
| **GGT (IU/L)** | 67 (37-139) |
| **Platelet (x10^9^/L)** | 221 (178-265) |

*Values are median (interquartile range), or number (%)*

**Supplementary Table 10: MICA rs2596542 and DEPDC5 rs1012068 and risk of HCC.**

| **Variant** | **CHC**  **(n=1501)** | **HCC**  **(n=188)** | **p-value** |
| --- | --- | --- | --- |
| ***MICA* rs2596542** |  |  |  |
| CC | 535 (35.6) | 78 (41.6) | 0.2 |
| CT | 697 (46.4) | 83 (44.1) |  |
| TT | 269 (18) | 27 (14.3) |  |
| ***DEPDC5* rs1012068** |  |  |  |
| TT | 791 (52.7) | 102 (54.3) | 0.3 |
| TG | 580 (38.6) | 65 (34.5) |  |
| GG | 130 (8.7) | 21(11.2) |  |

*The frequency (%) of MICA rs2596542 and DEPDC5 rs1012068 genotype in the entire CHC cohort (n=1501) and in a cohort of patients with HCC (n=188).* *Fisher-exact tests were used for comparison of frequency data between groups.*
